# Analysis of the Impact of Medical Features and Risk Prediction of Acute Kidney Injury for Critical Patients Using Temporal Electronic Health Record Data With Attention-Based Neural Network

**DOI:** 10.3389/fmed.2021.658665

**Published:** 2021-06-04

**Authors:** Zhimeng Chen, Ming Chen, Xuri Sun, Xieli Guo, Qiuna Li, Yinqiong Huang, Yuren Zhang, Lianwei Wu, Yu Liu, Jinting Xu, Yuming Fang, Xiahong Lin

**Affiliations:** ^1^Information Department, Fujian Province Lianpu Network Technology Co., Ltd., Quanzhou, China; ^2^Department of Nephrology, Jinjiang Municipal Hospital, Quanzhou, China; ^3^Department of Endocrinology, The Second Affiliated Hospital of Fujian Medical University, Quanzhou, China; ^4^School of Informatics, Xiamen University, Xiamen, China; ^5^Network and Information Technology Center, Sun Yat-sen University, Guangzhou, China; ^6^The Department of Endocrinology of the Seventh Affiliated Hospital of Sun Yat-sen University, Shenzhen, China

**Keywords:** acute kidney injury, medical features impact, electronic health record data, temporal convolutional network, attention based neural network

## Abstract

Acute kidney injury (AKI) is one of the most severe consequences of kidney injury, and it will also cause or aggravate the complications by the fast decline of kidney excretory function. Accurate AKI prediction, including the AKI case, AKI stage, and AKI onset time interval, can provide adequate support for effective interventions. Besides, discovering how the medical features affect the AKI result may also provide supporting information for disease treatment. An attention-based temporal neural network approach was employed in this study for AKI prediction and for the analysis of the impact of medical features from temporal electronic health record (EHR) data of patients before AKI diagnosis. We used the publicly available dataset provided by the Medical Information Mart for Intensive Care (MIMIC) for model training, validation, and testing, and then the model was applied in clinical practice. The improvement of AKI case prediction is around 5% AUC (area under the receiver operating characteristic curve), and the AUC value of AKI stage prediction on AKI stage 3 is over 82%. We also analyzed the data by two steps: the associations between the medical features and the AKI case (positive or inverse) and the extent of the impact of medical features on AKI prediction result. It shows that features, such as lactate, glucose, creatinine, blood urea nitrogen (BUN), prothrombin time (PT), and partial thromboplastin time (PTT), are positively associated with the AKI case, while there are inverse associations between the AKI case and features such as platelet, hemoglobin, hematocrit, urine, and international normalized ratio (INR). The laboratory test features such as urine, glucose, creatinine, sodium, and blood urea nitrogen and the medication features such as nonsteroidal anti-inflammatory drugs, agents acting on the renin–angiotensin system, and lipid-lowering medication were detected to have higher weights than other features in the proposed model, which may imply that these features have a great impact on the AKI case.

## Introduction

Acute kidney injury (AKI) refers to a sudden or sustained decline in renal function, clinically manifested as azotemia, water electrolyte and acid–base balance disorders, and systemic symptoms, accompanied by oliguria or anuria (1). AKI is very common among hospitalized patients in the intensive care unit (ICU), with an incidence of up to 57.3% (2). Once AKI occurs, the length of hospital stay, medical burden, incidence of chronic kidney disease, and mortality increase significantly (3). Early identification and intervention are the keys to improve the prognosis of AKI patients. Since the factors that lead to AKI are complex, statistical, or machine learning algorithms are used to analyze the important pathogenic factors and build risk assessment models based on various electronic health record (EHR) data, which is currently an important approach for the early detection and prognosis analysis of AKI (4).

Studies of training EHR data with machine learning technologies have shown great potential on clinical research, clinical decision-making, and disease prediction. Rough et al. (5) used the long short-term memory (LSTM) model to predict inpatient medication orders from EHRs. Yang et al. (6) predicted discharge medications at admission time based on the convolutional neural network (CNN). Miotto et al. (7) considered comprehensive data of patient and predicted the future patients from EHR data using the random forest model. Darabi et al. (8) proposed a time-aware patient representation method from EHR data based on the feedforward neural network (FNN). Choi et al. (9) extracted clinical diagnosis codes as base data and used recurrent neural network models for early detection of heart failure onset. Nguyen et al. (10) constructed a convolutional net to represent patient features from medical records.

There are also some studies using data-driven technologies on AKI prediction. For example, Li et al. (11) applied NLP (12) to clinical notes and extracted meaningful features on early prediction on AKI. Tomašev et al. (13) used clinical data to predict a time course of the probability that a patient will develop AKI based on recurrent neural network (RNN). Xu et al. (14) identified sub-phenotypes of AKI using structured and unstructured data with memory network. Koyner et al. (15) developed a machine learning inpatient acute kidney injury prediction model by EHR data.

However, these methods focus on building a neural network model to predict the AKI case since admission to the hospital, but little about the prediction of the AKI stage and accurate onset time interval of AKI and analysis about how the medical features affect the AKI result.

In our study, we propose a temporal convolutional network to predict the future value of the temporal data such as lab test and vital sign from EHR data, and then we use an attention-based model which combines these predicted future values with other features of the patient, such as demographic data, admission diagnosis code, and medication codes, as the input of the model, to make the AKI prediction and analyze the impact of each selected medical feature.

The main contributions of our work can be summarized as follows:

- An attention-based neural network model is proposed to improve the prediction performance of the AKI case with improvement of around 5% AUC, compared with the recent AKI prediction approaches—memory networks (MN) and hierarchical LSTM (HieLSTM) (14).
- The proposed model is capable of predicting the AKI stage and onset time interval which are meaningful in clinical practice.
- We explore the associations between medical features and the AKI case and the impact of medical features to AKI prediction result, which may help improve treatment.
- The proposed model has been applied in clinical practice, and its performance has been remarkable.

## Materials and Methods

### AKI Definition

#### AKI Criteria

There are four criteria used for AKI diagnosis: the Risk-Injury-Failure-Loss-End (RIFLE) criteria (16), the pediatric RIFLE (pRIFLE) criteria (17), the Acute Kidney Injury Network (AKIN) criteria (18), and the Kidney Disease: Improving Global Outcomes (KDIGO) criteria (19), and these diagnosis criteria are all based on patients' serum creatinine (SCr) and urine volume. Because KDIGO is widely used for both AKI research and clinical diagnosis, we take the KDIGO criteria to define the AKI case and stages.

#### AKI Case

Based on the definition provided by the KDIGO criteria, an AKI case can be identified by any one of the following conditions:

- SCr increases by ≥0.3 mg/dl (26.5 mol/L) within 48 h.
- SCr increases ≥1.5 times from the baseline that comes from the first SCr value measured during hospitalization within 7 days.
- Urine volume is <0.5 ml/kg/h for 6 h.

#### AKI Stages

We used the definition provided by the KDIGO criteria for AKI stages (19) described as follows:

- Stage 1: SCr is 1.5–1.9 times from the baseline, which comes from the first SCr value measured during hospitalization within the prior 7 days; or SCr increases by ≥0.3 mg/dl (26.5 mol/L) within 48 h; or urine volume is <0.5 ml/kg/h for 6–12 h.
- Stage 2: SCr increases to ≥2.0–2.9 times from the baseline, which comes from the first SCr value measured during hospitalization within the prior 7 days, or urine volume is <0.5 ml/kg/h for ≥12 h.
- Stage 3: SCr increases to ≥3.0 times from the baseline, which comes from the first SCr value measured during hospitalization within the prior 7 days; or SCr increases by ≥4.0 mg/dl (353.6 mol/L) within 48 h; or initiation of renal replacement therapy; or urine volume is <0.3 ml/kg/h for ≥24 h; or anuria for ≥12 h; or in patients <18 years, a decrease in eGFR (20) to <35 ml/min per 1.73 m^2^.

#### AKI Onset Time Interval

We define the observation interval as the duration elapsed from the entrance time of the ICU to a certain time point *T*_observ_end_ (this time point is referred to as the end time of the observation interval), and the prediction interval is defined as the duration elapsed from the end time of the observation interval to a certain time point *T*_predict_end_ (this time point is referred to as the end time of the prediction interval). The prediction interval will be divided into several subintervals, and each subinterval has the same time length. We name this subinterval as the onset interval which is referred to as the AKI onset time interval, and our model tries to predict the accurate AKI onset interval in patients.

### Data Preparation

#### Data Source

We use the Medical Information Mart for Intensive Care III (MIMIC-III) database (21) for model training, validation, and testing. There were 46,520 patients, 58,976 admission records, and 61,532 records of intensive care unit (ICU) stay from 2001 to 2012. The patient information contained in this database includes patient demographics, vital signs, laboratory test results, procedures, medications, clinical notes, imaging reports, and patient mortality.

#### Patient Features

The patient features we considered into the proposed model can be classified as follows:

- Demographics: gender, age, and ethnicity of the patients.
- Body mass index (BMI) data: mass (in kilograms), height (in meters), BMI value (calculated by mass/height^2^).
- Vital signs: in this group, we take the following features in the proposed model: blood pressure (including diastolic blood pressure, systolic blood pressure, and mean arterial blood pressure), blood oxygen saturation value, heart rate, respiration rate, glucose (both lab and fingerstick), and body temperature.
- Laboratory test results: as an important part of the patients' features, the following biochemical criteria are considered as the model input: serum creatinine, total urine volume in the first 24 h of ICU stay, anion gap, albumin, bands, bilirubin, hematocrit, lactate, sodium, bicarbonate, blood urea nitrogen (BUN), calcium, chloride, creatinine, hemoglobin, international normalized ratio (INR), platelet, potassium, prothrombin time (PT), partial thromboplastin time (PTT), and white blood count (WBC).
- Medications: patients' medication records during the admission. In our study, according to the Anatomical Therapeutic Chemical (ATC) Classification System (22), we focus mainly on the following categories: drugs used in diabetes, antithrombotic agents, antihypertensives, diuretics, agents acting on the renin–angiotensin system, lipid-lowering medication, non-steroidal anti-inflammatory drugs (NSAIDs), and contrast media.
- Comorbidities: for comorbidities of the patients that may affect the AKI result, we get the comorbidity information from the admission diagnosis notes since we cannot use the data after the prediction time, and the following keywords are used to retrieve information from patients' admission notes: congestive heart failure, peripheral vascular, hypertension, diabetes, liver disease, myocardial infarction, coronary artery disease (CAD), cirrhosis, jaundice, sleep apnea, and urinary tract infection.

### Experimental Setup

In our study, each ICU stay record is considered as a data sample, and we take the KDIGO criteria to define the AKI case and AKI stages for the records.

Patients' data in the observation interval will be considered as the training data of the proposed model, and the data in the prediction interval are used to get the AKI results, including the AKI case, AKI stage, and AKI onset interval, according to the KDIGO criteria, as the output label of the model.

Our model is applied to three experimental cases by setting different elapsed times of observation interval, prediction interval, and onset interval as follows:

- Experimental case 1: we use patients' data during the first 24 h of ICU stay (the time length of observation interval is 24 h) to predict the AKI case, AKI stage, and AKI onset time interval in the next 24 h (the time length of prediction interval is 24 h), and we set the time length of onset interval to 12 h for our predictions.
- Experimental case 2: we use patients' data during the first 24 h of ICU stay (the time length of observation interval is 24 h) to predict the AKI case, AKI stage, and AKI onset interval in the next 6 days (the time length of prediction interval is 6 days), and we set the time length of onset interval to 24 h for our predictions.
- Experimental case 3: we use patients' data during the first 48 h of ICU stay (the time length of observation interval is 24 h) to predict the AKI case, AKI stage, and AKI onset interval in the next 5 days (the time length of prediction interval is 5 days), and we set the time length of onset interval to 24 h for our predictions.

**Figure 1:**
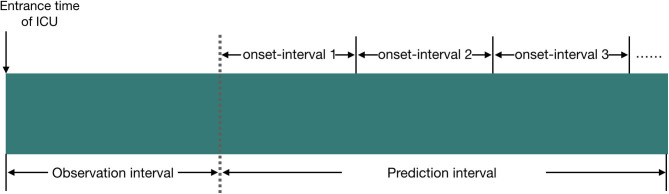
Illustration of the experiment setup in our study, and the relationship between observation interval, prediction interval, and onset interval. We set the time length of observation interval to 24 or 48 h; the time length of prediction interval to 24 h, 5 days, or 6 days; and the time length of onset interval to 12 or 24 h.

We provide more information about our experiment setup in Figure 1.

#### Data Filter

Since our main task is to predict the future AKI case, AKI stage, and AKI onset time interval for patients who are not AKI cases during the observation interval, and because patients' medical data are necessary for effective prediction, the following patient cases will be excluded from the dataset:

- Patients who had AKI diagnosis in their admission notes,
- Patients who did not have laboratory test results during the ICU stay,
- Patients who did not have the admission notes, and
- Patients who were admitted as AKI cases during the observation interval.

### Data Preprocessing

**Figure 2:**
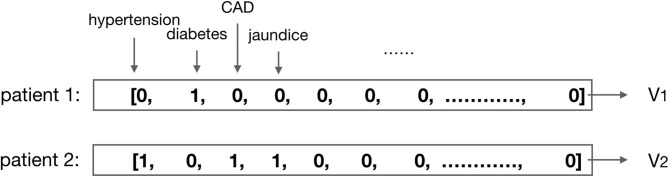
Illustration of the multi-hot vector representation for patients' comorbidities. For example, the comorbidity of patient 1 is diabetes, then vector V_1_ is the multi-hot vector representation for patient 1; the comorbidities of patient 2 are hypertension, CAD (coronary artery disease), and jaundice, then vector V_2_ is the multi-hot vector representation for patient 2.

Before the data training, we preprocess the data by the following ways:

1) Drug code mapping: the drug information provided in the MIMIC-III dataset was indexed by the National Drug Code (NDC) (23) which serves as a universal product identifier for drugs, published by the Food and Drug Administration (FDA) (24). Since we need to classify the drugs by their clinical usage, we map the NDC code to ATC code, and with the help of the ATC code, we retrieve the information of the categories.
2) Absent value process: for the missing values of the patients' features that we consider as the training data of the proposed model during the whole observation interval, we impute them by the default normal values.
3) Normalization process of medical results: to ensure the model training to be effective, we need to normalize the values. For the discrete values, such as gender, medication code, and comorbidities, the one-hot or multi-hot vector is employed for the representation (shown in Figure 2); for the continuous values, such as the laboratory test results and vital signs, we use the linear normalization function to do the normalization: *n*is the size of the dataset, *x* is the original value that needs to be normalized, and $\hat{x}$ is the normalized value according to *x*.
  (1) $$\begin{array}{r} \hat{x}=\frac{x-\underset{0\leq j\leq n}{\min}\{x_{j}\}}{\underset{0\leq j\leq n}{\max}\{x_{j}\}-\underset{0\leq j\leq n}{\min}\{x_{j}\}} \end{array}$$
4) Temporal value process of the medical results: for a specified feature, there may be multiple check values during the observation interval, which are meaningful to the prediction of the future value, and we create the vector to represent temporal value order by the checking time.

### The Predictive Models

The tasks of our study are the prediction of the AKI case, AKI stage, and AKI onset time interval by patients' temporal medical data during the ICU stay. We build the predictive models which take the patients' features that we described above as the input data to get an output vector which contains one or multiple probability value(s). For each value in the vector, we compare it with a chosen threshold: if the value in the vector exceeds the threshold, then it will be reset to 1, which means a positive prediction; otherwise, it will be reset to 0, which means a negative prediction.

**Figure 3:**
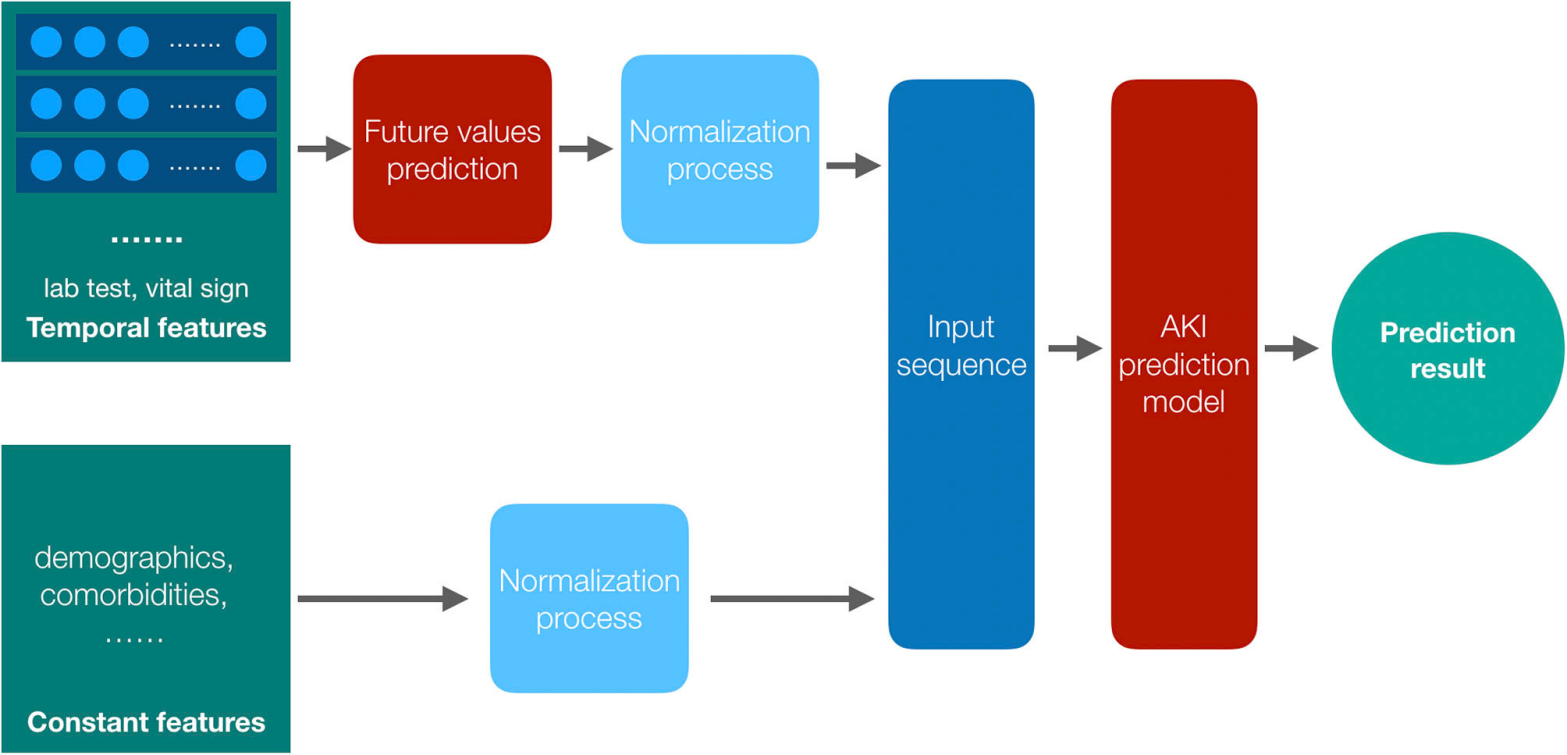
The framework of the prediction models. Both the temporal features and the constant features are preprocessed before training, and we get the future values for the temporal features by their historical data; then, the two parts of the input data, values of constant features and the future values of temporal features, are combined together as the input data of the AKI prediction model to get the result.

We classify the patients' feature data into two categories: constant features such as demographics and temporal features such as the laboratory test results and the vital signs. We get the future values of the temporal features by a temporal convolutional network (TCN) model (25) and combine the constant features with these future values as the input sequence of the proposed models after the normalization process. With the AKI prediction model, we get the final results. The framework of the prediction models is shown in Figure 3.

#### Prediction of Future Values

The values of the temporal features play important roles in our AKI prediction, especially the laboratory test results and vital signs. The future value of each feature may help improve the prediction performance, and TCN is employed to predict the future values according to historical data for its superior performance on the prediction of time series data and the variable length of the input sequence.

TCN is a convolutional network which convolves over the time domain (26), and it is trained to predict the future values for the input time series. There are two main principles of TCN: the output of the network should have the same length as its input, and the network can only use the information from past time steps (27). Suppose the input sequence of the model is *x*_0_, *x*_1_, …, *x*_*T*_, and with the TCN model network *F*, we get the output sequence *y*_0_, *y*_1_, ..., *y*_*T*_:

(2) $$\begin{array}{r} y_{0},y_{1},\ldots ,y_{T}=F\left(x_{0},x_{1},\ldots ,x_{T}\right) \end{array}$$

The length of the output sequence is the same as that of the input sequence, and the value *y*_*t*_ only depends on the sequence *x*_0_, *x*_1_, …, *x*_*t*_, which satisfies the two principles mentioned above.

TCN uses causal convolutions, which make an output at time *t* to convolve only with elements from time *t* and earlier in the previous layer (28). However, a simple causal convolution is only able to look back at history with size linear in the depth of the network (28), which makes causal convolution to have a poor performance on the prediction of sequence tasks that require long history. To resolve the issue, dilated convolutions are employed in TCN. Suppose *X* is the input sequence, and *X* ∈ ℝ^*n*^, where *n* is the length of the input sequence, and we have a filter: *f*:{0, ..., *k* − 1} → ℝ, then the process of the dilated convolution function *F*_*v*_ on element *v* of the input sequence *X* can be defined as:

(3) $$\begin{array}{r} F_{v}=\overset{k-1}{\underset{i=0}{\sum }}f\left(i\right)\cdot X_{v-d\cdot i} \end{array}$$

**Figure 4:**
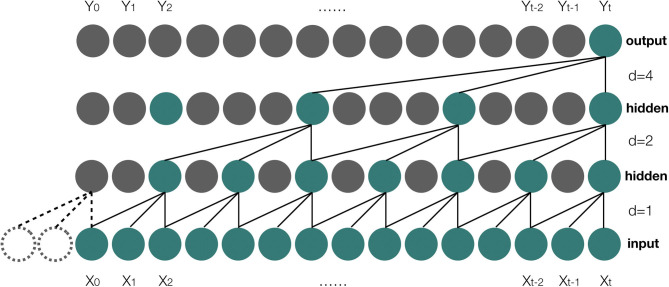
A dilated casual convolution with dilated factors d = 1, 2, 4 and filter size k = 3. X_0_, X_1_, …, X_t_ are the elements of the input sequence, and Y_0_, Y_1_, …, Y_t_ are the elements of the output result of the model.

Where *d* is the dilation factor, *k* is the size of filter, while *v* − *d*·*i* gets the past index of the input sequence. The illustration about the architecture of the dilated causal convolution is shown in Figure 4.

In our study, the temporal values of each feature of the patients during the ICU stay are sorted by check time in ascending order after the normalization process, and these temporal values are considered as the input sequence of the TCN model. We get the last value of the output sequence as the predicted future value which will be used later in the AKI prediction model.

#### The AKI Prediction Model

**Figure 5:**
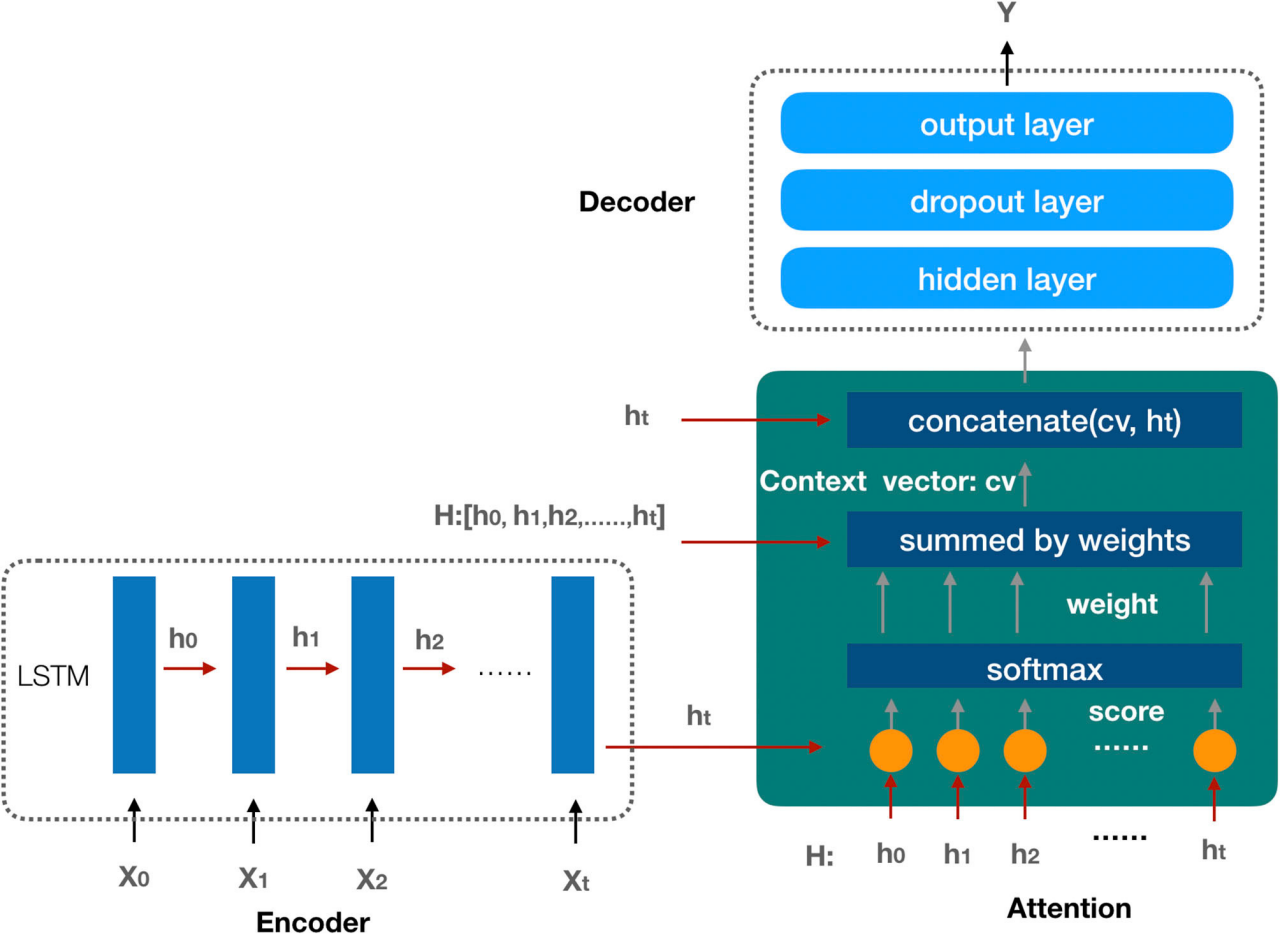
The architecture of the AKI prediction model. The encoder model is used to embed the input sequence X_0_, X_1_, …, X_t_ and get the hidden state in each recurrent unit by LSTM. All the hidden states are taken as the input of the attention function to create the context vector; finally, with the input context vector, the decoder model gets the prediction result.

Since the future AKI prediction results depend on patients' features data during the observation interval, and each feature has a different degree of influence on the AKI result, we employ the attention-based neural network (29) model as our AKI prediction model in our study. The prediction model is composed of three components: the encoder model, the attention function, and the decoder model (the architecture of the prediction model is shown in Figure 5).

##### Encoder Model

The encoder model is composed of several recurrent units to embed the input sequence. The LSTM (30) network is employed as the recurrent unit to process the input sequence, and the elements in the input sequence represent the last states (or the predicted future values) of the patient's selected features, such as demographics, vital signs, laboratory test results, medications, and comorbidities mentioned above. Each element of the input sequence *X*_*i*_ is processed by a LSTM unit to get the hidden state vector *h*_*i*_ which will be considered as the input of the next LSTM unit. After the process of the encoder model, we get a matrix *H* which is composed of all the hidden states. Suppose there are *t* + 1 recurrent units in the encoder model, then matrix *H* can be presented as follows:

(4) $$\begin{array}{r} H=\left[h_{0},h_{1},\ldots \ldots ,h_{t}\right] \end{array}$$

##### Attention Function

In our attention function, each hidden state *h*_*i*_ (0 ≤ *i* ≤ *t, t* + 1 is the number of hidden states) from the LSTM units of the encoder model is used to calculate the similarity score with the last hidden state *h*_*t*_ by the following formula:

(5) $$\begin{array}{r} f\text{\_}score\left(h_{t},h_{i}\right)=h_{t}\cdot h_{i} \end{array}$$

The function *f*_*score* computes the dot product of *h*_*t*_ with *h*_*i*_ as the similarity score *s*_*i*_, and then the *Softmax* function is employed to get the weight value *w*_*i*_ for each score *s*_*i*_ (the detail of the formula is shown as follows).

(6) $$\begin{array}{r} w_{i}=Softmax\left(s_{i}\right)=\frac{\exp\left(s_{i}\right)}{\sum_{j=0}^{t}\exp\left(s_{j}\right)} \end{array}$$

The attention context vector *cv* is generated by the sum of each hidden state *h*_*i*_ with their weight *w*_*i*_,

(7) $$\begin{array}{r} cv=\overset{t}{\underset{i=0}{\sum }}w_{i}^{*}h_{i} \end{array}$$

The context vector *cv* is finally concatenated with the last hidden state *h*_*t*_ as the output of the attention function *av*:

(8) $$\begin{array}{r} av=concatenate\left(cv,h_{t}\right) \end{array}$$

With the attention function, we transform the relationship between the patient's features and AKI into the vector representation *av* which will be applied as the input data of the decoder model.

##### Decoder Model

The decoder model is used for the AKI prediction by a three-layer neural network with the attention output vector *av* as the model input sequence. The first hidden layer is a full connected layer that transfers the feature information from the input sequence to the next layer. The dropout layer is employed in the second layer to improve the generalization of the model by randomly setting the output of a given neuron to 0 at each update of the training phase (31). In the output layer, because of the different prediction tasks, we consider the following situations:

1) AKI case prediction: since the AKI case prediction is a binary classification task that detects if the given sample is an AKI case, we employ the function sigmoid as the active function to get the prediction value that is in the interval [0,1]:
  (9) $$\begin{array}{r} Sigmoid\left(x\right)=\frac{1}{1+exp\left(-x\right)} \end{array}$$
2) AKI stage prediction: according to the AKI stage definition, there are three AKI stages, and we employ the function *Softmax* as the active function (details about the *Softmax* function are described in Equation 7). The output is a three-dimensional vector, and each value in the vector represents the probability of the corresponding AKI stage.
3) AKI onset time interval prediction: the AKI onset time interval prediction is a multi-class classification task that detects the accuracy time interval of the AKI case, similar with the AKI stage prediction, and the function *Softmax* is used as the active function of this layer to get the result. As mentioned above, we have three experimental cases, and the dimension of the output vector is set to be the number of the onset intervals in each experimental case.

#### Parameter Settings

1) In the TCN model, we employ a three-layer neural network for the data training. The value of the dilation factor in the first hidden layer is set to be 1, while it is 2 in the second hidden layer, and 4 in the output layer. The size of the filter is set to 3 and the hidden units are set to 16. The function ReLU (32) is used as the active function, and the Adam optimization scheme (33) is used as the optimizer of the model. The learning rate is set to 0.001, and we have 100 epochs for the model training.
2) In the AKI prediction model, the dimension of the input sequence is 50 according to the patient features we selected, so there are 50 recurrent units in the encoder model. We set the output dimension of each unit to 64; therefore, the dimension of the context vector is 64, and the dimension of the attention function is 128 after the concatenation of the context vector with the last hidden state of the encoder model. In the decoder model, there are 128 hidden units in the first hidden layer. The dropout value is set to 0.2 in the second hidden layer, while it has the same hidden units as the first layer. The initial learning rate is 0.001 with a decay factor of 0.9, and we use the Adam optimization scheme as the optimizer during the data training. There are 1,000 epochs and the batch size is 128 in each epoch.

### Baseline Methods

To validate the performance of the proposed model, we employed the gradient boosted trees (GBTs) (34), logistic regression (LR) (35), random forest (RF) (36), and LSTM (37) as the AKI case prediction methods to compare with the proposed model. Besides, we also had a comparison between the AKI prediction with future predicted values and that without future predicted values. We implemented these methods in the three experimental cases mentioned above to get their performances on AKI case prediction, AKI stage prediction, and AKI onset time interval prediction. As the input data of the model, we considered demographics data, laboratory test results, vital signs, medications, and comorbidities. For the continuous data, we used the linear normalization function to ensure them in [0, 1], and for the discrete data, we used one-hot or multi-hot to map them into a vector. To avoid leaking future information to the models, we only took the features data during the observation interval, without any data in the prediction interval.

The proposed model was implemented in Python 3.6, with TensorFlow 2.3.0 (38) as the deep learning library, and the code was running on a server with NVIDIA Tesla P10 GPU. The machine learning library scikit-learn (39) was employed for LR, RF, and GBT implementations.

## Results

### Prediction Results on MIMIC-III Dataset

In our study, we created three experimental cases, and the dataset in each case was divided into three sets, namely, training, validation, and testing sets, according to the ratio of 8:1:1. In order to achieve robust performances on the predictions of AKI case, AKI stage, and AKI onset time interval, each of the following ratios remained consistent in the three sets: ratio of AKI cases to not AKI cases, ratio of patients' number in each AKI stage, and ratio of patients' number in each onset interval.

In each of the three experimental cases, we firstly applied our models together with the baseline models mentioned in *Baseline methods* to get the performance of AKI case prediction by specificity, sensitivity, and AUC (area under the receiver operating characteristic curve), and then the proposed model was implemented to get the performances of AKI stage prediction and AKI onset time interval prediction. The performance of each prediction task is shown as average ± standard deviation (the results of the experimental cases are shown in Tables 1–3).

**Table 1:** Acute kidney injury (AKI) case prediction: the performance of the methods applied in the experimental cases.

**Experimental case**	**Methods**	**Specificity**	**Sensitivity**	**AUC**
Case 1	AM with PFV	0.7612 ± 0.0121	0.7401 ± 0.0117	0.8320 ± 0.0128
Case 1	AM without PFV	0.6987 ± 0.0109	0.7242 ± 0.0134	0.8109 ± 0.0103
Case 1	LSTM	0.6237 ± 0.0112	0.6808 ± 0.0129	0.7586 ± 0.0154
Case 1	GBTs	0.5631 ± 0.0093	0.6591 ± 0.0107	0.7323 ± 0.0140
Case 1	RF	0.4909 ± 0.0145	0.6214 ± 0.0178	0.7190 ± 0.0167
Case 1	LR	0.4012 ± 0.0119	0.6022 ± 0.0201	0.7078 ± 0.0198
Case 2	AM with PFV	0.7261 ± 0.0130	0.7527 ± 0.0190	0.8260 ± 0.0108
Case 2	AM without PFV	0.6704 ± 0.0145	0.7358 ± 0.0125	0.8045 ± 0.0110
Case 2	LSTM	0.5409 ± 0.0119	0.7167 ± 0.0140	0.7591 ± 0.0169
Case 2	GBTs	0.5033 ± 0.0121	0.6559 ± 0.0138	0.7183 ± 0.0136
Case 2	RF	0.4597 ± 0.0172	0.6371 ± 0.0117	0.6992 ± 0.0171
Case 2	LR	0.4143 ± 0.0131	0.6120 ± 0.0193	0.6790 ± 0.0126
Case 3	AM with PFV	0.7921 ± 0.0112	0.76231 ± 0.0127	0.85751 ± 0.0153
Case 3	AM without PFV	0.7528 ± 0.0177	0.7367 ± 0.0116	0.8229 ± 0.0199
Case 3	LSTM	0.6268 ± 0.0105	0.7019 ± 0.0182	0.7783 ± 0.0165
Case 3	GBTs	0.5775 ± 0.0165	0.6644 ± 0.0155	0.7408 ± 0.0106
Case 3	RF	0.5101 ± 0.0109	0.6499 ± 0.0181	0.7387 ± 0.0101
Case 3	LR	0.4243 ± 0.0113	0.6231 ± 0.0130	0.7199 ± 0.0160

*AM, attention model; PFV, predicted future values by TCN; LTSM, long short-term memory; GBTs, gradient boosted trees; RL, logistic regression; RF, random forest*.

**Table 2:** AKI stage prediction: the AKI stage prediction performance of the attention-based model with predicted future values in the experimental cases.

**Experimental case**	**AKI stage**	**Specificity**	**Sensitivity**	**AUC**
Case 1	Stage 1	0.5235 ± 0.0143	0.7928 ± 0.0180	0.7309 ± 0.0144
Case 1	Stage 2	0.5563 ± 0.0115	0.8066 ± 0.0109	0.7874 ± 0.0129
Case 1	Stage 3	0.7720 ± 0.0118	0.7991 ± 0.0203	0.8671 ± 0.0107
Case 2	Stage 1	0.5057 ± 0.0161	0.7651 ± 0.0145	0.6964 ± 0.0126
Case 2	Stage 2	0.5387 ± 0.0124	0.7703 ± 0.0172	0.7387 ± 0.0174
Case 2	Stage 3	0.6992 ± 0.0181	0.7914 ± 0.0188	0.8264 ± 0.0152
Case 3	Stage 1	0.5416 ± 0.0119	0.7139 ± 0.0107	0.6742 ± 0.0097
Case 3	Stage 2	0.5837 ± 0.0143	0.8083 ± 0.0128	0.7655 ± 0.0130
Case 3	Stage 3	0.7302 ± 0.0149	0.7751 ± 0.0139	0.8279 ± 0.0081

**Table 3:** AKI onset time interval prediction: the AKI onset time interval prediction performance of the attention-based model with predicted future values in the experimental cases.

**Experimental case**	**AKI onset interval**	**Specificity**	**Sensitivity**	**AUC**
Case 1	0–12 h	0.5266 ± 0.0097	0.5937 ± 0.0113	0.6891 ± 0.0104
Case 1	12–24 h	0.5049 ± 0.0131	0.5791 ± 0.0099	0.6750 ± 0.0119
Case 2	0–24 h (day 1)	0.5681 ± 0.0109	0.7197 ± 0.0122	0.7318 ± 0.0141
Case 2	24–48 h (day 2)	0.5373 ± 0.0138	0.6786 ± 0.0108	0.7035 ± 0.0159
Case 2	48–72 h (day 3)	0.3928 ± 0.0101	0.6090 ± 0.0137	0.6271 ± 0.0190
Case 2	72–96 h (day 4)	0.2049 ± 0.0113	0.5182 ± 0.0109	0.5319 ± 0.0138
Case 2	96–120 h (day 5)	0.1828 ± 0.0122	0.4901 ± 0.0117	0.5193 ± 0.0112
Case 2	120–144 h (day 6)	0.1528 ± 0.0140	0.4123 ± 0.0159	0.5042 ± 0.0112
Case 3	0–24 h (day 1)	0.5865 ± 0.0116	0.7291 ± 0.0091	0.7518 ± 0.0131
Case 3	24–48 h (day 2)	0.5448 ± 0.0128	0.6890 ± 0.0085	0.7091 ± 0.0106
Case 3	48–72 h (day 3)	0.4028 ± 0.0079	0.6190 ± 0.0167	0.6371 ± 0.0159
Case 3	72–96 h (day 4)	0.2019 ± 0.0108	0.5149 ± 0.0149	0.5279 ± 0.0171
Case 3	96–120 h (day 5)	0.1799 ± 0.0113	0.5011 ± 0.0132	0.5128 ± 0.0110

From these results, we observe that:

1) The performances of the deep learning models were better than the machine learning algorithms on the AKI case prediction, and this result may be explained by the fact that the deep learning model can better obtain the feature dependencies among the EHR data, which could be beneficial to the AKI case prediction.
2) Compared with the LSTM model, our proposed model improved the performance on the AKI case prediction by around 7% AUC, and this discrepancy could be attributed to the attention mechanism that we employ in the proposed model, which can learn the influence degree of each feature to the AKI case in the model.
3) Compared with the recent AKI case prediction approaches, such as MN and HieLSTM (14) which achieved 77.53% AUC by 24-h observation interval and 77.98% AUC by 48-h observation interval, our model improved the prediction performance to 82.60% AUC by 24-h observation interval and 85.75% AUC by 48-h observation interval (shown in Table 1).
4) In the proposed model, we compared the attention-based model with predicted future values (AM with PFV) to that without predicted future values (AM without PFV), and the improvement of the AKI case prediction performances by AM with PFV was around 4% AUC. The difference between the two models was the temporal feature value of the input sequence: for each temporal feature, the model AM with PFV used the predicted future value, which was provided by the TCN model, as the element value of the input sequence, while the model AM without PFV chose the last value from historical data. Since the predicted future value can be interpreted as the combination of the last value and the value variation in the future, it may better reflect the health trend of the patient than the last value of the historical data, and this may be the response for the different performances between the two models.
5) The performance of the AKI case prediction with the proposed model in experimental case 2 was around 82% AUC, while it was around 85% AUC in experimental case 3. Since the observation interval in case 2 was 24 h and that in case 3 was 48 h, more temporal features data in case 3 were considered into the proposed model to better learn the trend of the AKI risk.
6) For the AKI onset time interval prediction, we found that in experimental case 2 and case 3, the prediction performance of the proposed model was around 70% AUC in the first two onset intervals (the length of each onset interval is 24 h) of the prediction interval, but it came to around 52% AUC in the remaining onset intervals.

### Performance in Clinical Practice

The proposed model was applied to clinical AKI prediction for ICU patients, and we took the value of the selected features during the first 24 h of the patients' ICU stay, to predict the AKI risk in the next 6 days, including the prediction of the AKI case, AKI stage, and AKI onset time interval (24 h as the length of each onset interval).

Before we apply the model for AKI prediction, we processed the clinical data as follows:

1) The data structure transform: the initial clinical data were exported from the hospital's information system and saved in an Excel file. We designed a script to parse the data in the exported file, and data were imported to our database by a structure that is compatible with the model's data loader.
2) Comorbidity label design: since the admission diagnosis notes in clinical data were recorded in Chinese, to get the comorbidity information, lists of Chinese keywords were provided to retrieve the information.
3) Data verification: the filter conditions (details in sections Data Filter and Experimental Setup) were implemented on the data to exclude invalid patients' data.
4) Detail preprocessing: like the data preprocessing on MIMIC-III data (details in the section Data Preprocessing), we also applied it on clinical data, to preprocess the details.

**Figure 6:**
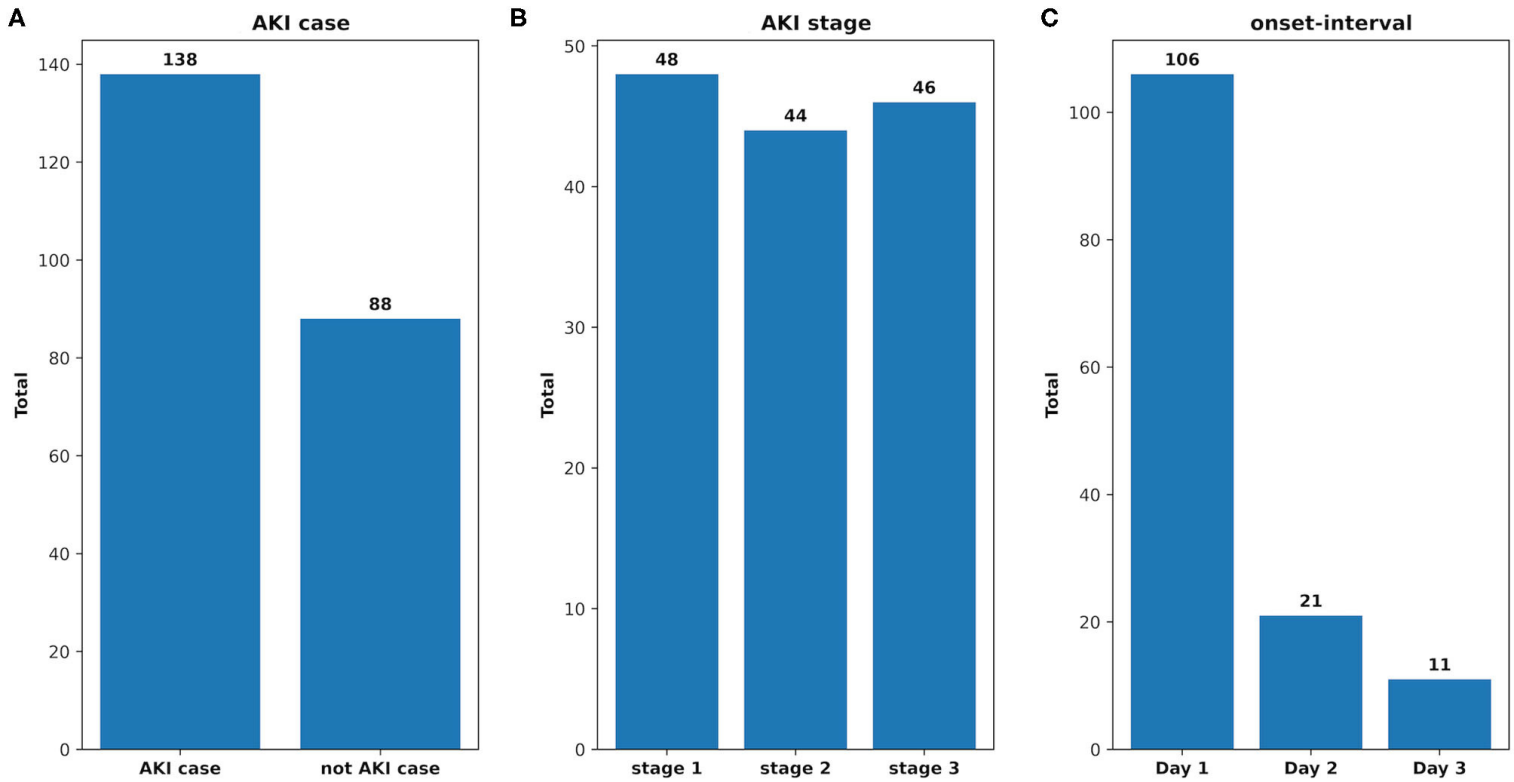
The analysis of the clinical dataset. **(A)** AKI case: the total number of the patients who developed AKI and that of the not AKI case; **(B)** AKI stage: the total number of the AKI patients in each stage; **(C)** onset interval: the total number of AKI patients in each onset interval.

**Figure 7:**
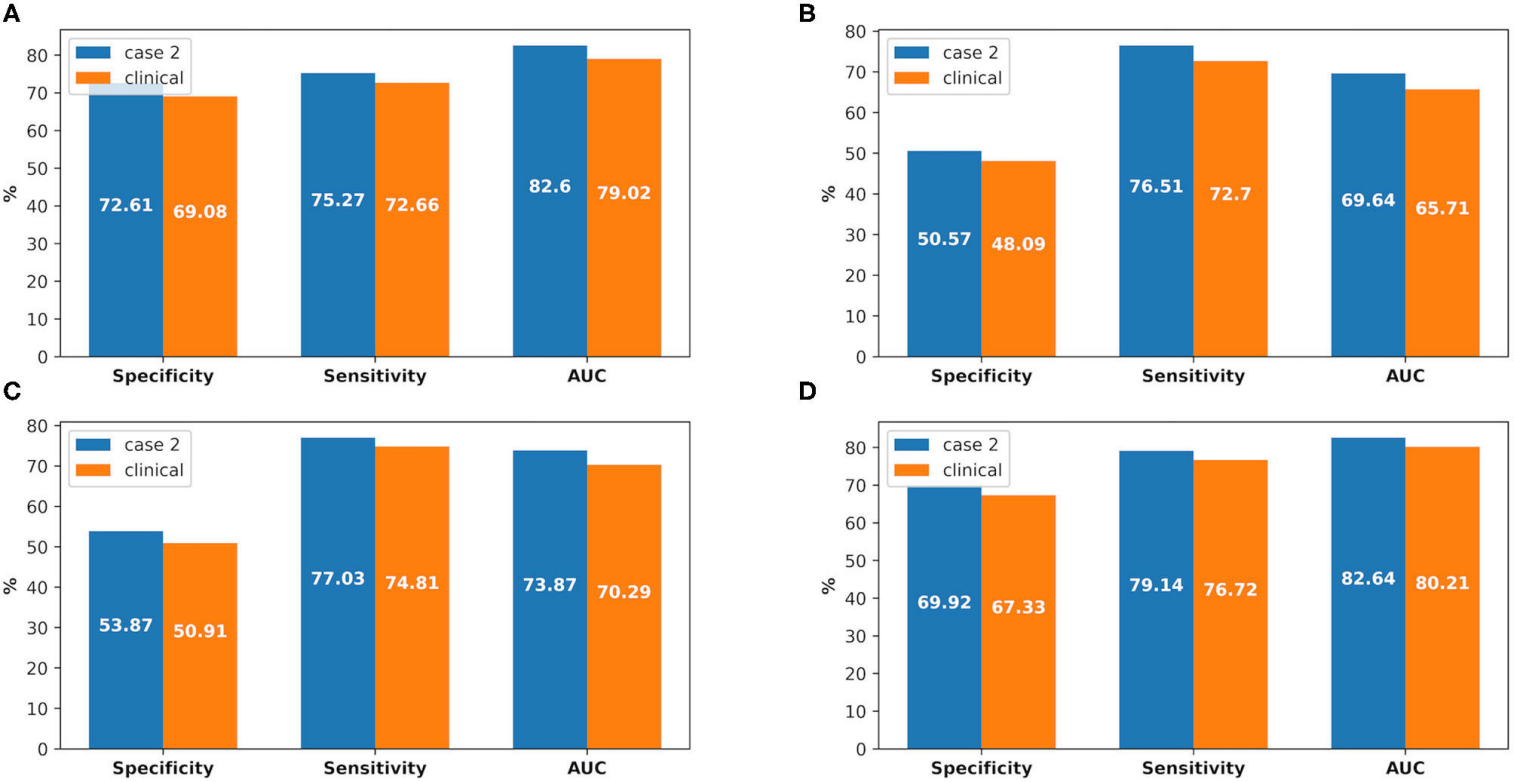
The comparison between experimental case 2 and clinical practice, at AKI case prediction and AKI stage prediction. **(A)** AKI case prediction, **(B)** AKI stage 1 prediction, **(C)** AKI stage 2 prediction, **(D)** AKI stage 3 prediction.

**Figure 8:**
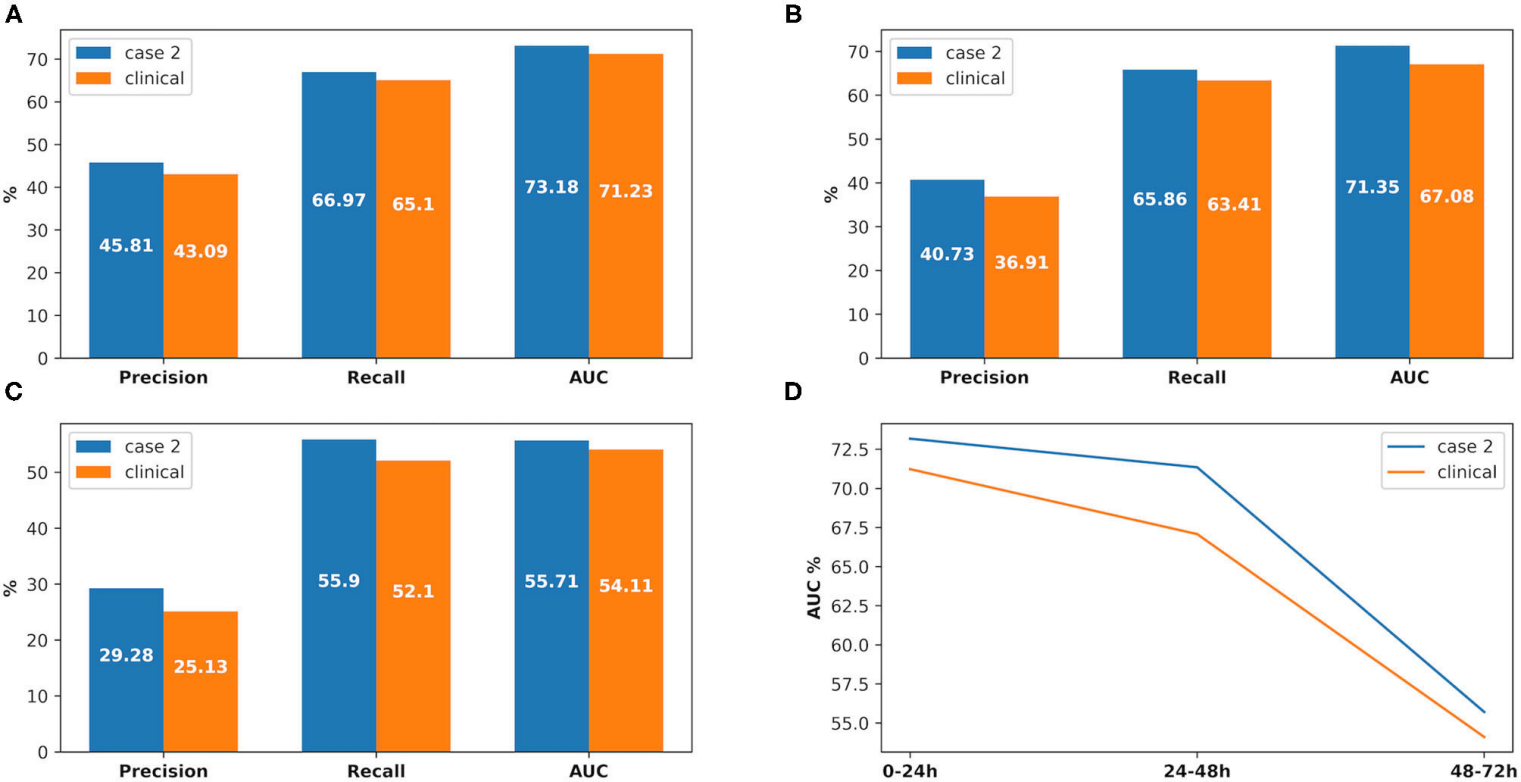
The comparison between experimental case 2 and clinical practice, at AKI onset time interval prediction. **(A)** Prediction in 0–24 h, **(B)** prediction in 24–48 h, **(C)** prediction in 48–72 h, **(D)** comparison between the onset time intervals.

There were 226 patients tested by the model, and details of the dataset are shown in Figure 6. The comparison of the model performance between experimental case 2 and clinical practice is shown in Figure 7 and summarized as follows:

1) The prediction performance of AKI case in clinical practice was around 3.5% less on specificity, while it was around 2.6% less on sensitivity and 3.6% less on AUC (shown in Figure 7A).
2) The prediction performance of AKI stage 1 in clinical practice was around 4% less on AUC, while it was around 3.6% less on AUC for stage 2 and 2.4% less on AUC for stage 3 (shown in Figures 7B–D).
3) The prediction performance of AKI onset time interval in clinical practice was around 3% less on AUC in the first onset interval (0–24 h), while it was around 6.3% AUC less in the second onset interval (24–48 h) and 7.6% AUC less in the third one (48–72 h). The details are shown in Figure 8.

### Analysis of the Impact of Medical Features on AKI

To further discover how the medical features in our study impact on AKI, we analyzed the selected features data in our study, including laboratory test results, vital signs, medications, and comorbidities, to identify their different representations between AKI cases and not AKI cases. The analysis was composed of two steps: firstly, we analyzed the association between the selected medical features and the AKI results; secondly, we detected the impact of weights of each medical feature with the help of the attention-based neural network. The analysis was based on the dataset of experimental case 1, which included 46,385 patients, consisting of 13,935 AKI cases and 32,450 not AKI cases.

#### Association Analysis

We firstly analyzed the laboratory test results and vital signs between the AKI case and not AKI case (the analysis result is shown in Table 4) to discover the association between each feature and the AKI result, positive or inverse. From the table, we find that several values in the last column are higher than 15%, such as lactate, glucose, creatinine, blood urea nitrogen, prothrombin time, and partial thromboplastin time, which may show that there are positive associations between the AKI case and these features; besides, there are also some values in the last column which are lower than −15%, such as platelet, hemoglobin, hematocrit, urine, and INR, which may reflect that they are inversely associated with the AKI case.

**Table 4:** Analysis of laboratory test results and vital signs by AKI case.

**Feature name**	**AKI**	**Not AKI**	**Delta: AKI–not AKI**	**Ratio: Delta/AKI (%)**
Lactate	0.0436	0.0235	0.0201	46.12
Creatinine	0.0683	0.0412	0.0271	39.69
BUN	0.1076	0.0755	0.0321	29.85
PT	0.0515	0.0365	0.015	29.17
PTT	0.1676	0.1256	0.042	25.08
Glucose	0.9883	0.8164	0.1719	17.39
Anion gap	0.2427	0.227	0.0158	6.50
Potassium	0.186	0.1774	0.0086	4.63
Bilirubin	0.0213	0.0205	0.0008	3.72
WBC	0.0199	0.0193	0.0007	3.28
Chloride	0.7187	0.713	0.0058	0.80
SpO_2_	0.968	0.9653	0.0027	0.28
Temperature	0.8281	0.8266	0.0015	0.18
Sodium	0.7522	0.7551	−0.0029	−0.38
SBP	0.4373	0.4462	−0.0089	−2.04
Bicarbonate	0.3947	0.4048	−0.0101	−2.57
Albumin	0.4383	0.4594	−0.021	−4.80
RR	0.2919	0.3065	−0.0146	−4.99
MBP	0.2513	0.2706	−0.0194	−7.70
Heart rate	0.3574	0.3892	−0.0318	−8.89
DBP	0.3221	0.3611	−0.0389	−12.08
Platelet	0.1189	0.1394	−0.0205	−17.25
Hemoglobin	0.3692	0.4411	−0.0719	−19.47
Hematocrit	0.3731	0.4458	−0.0728	−19.51
Urine	0.655	0.7905	−0.1355	−20.68
INR	0.0266	0.0351	−0.0085	−32.14

*The value in the column “AKI” represents the mean of each normalized feature value by AKI patients, while it is the mean of each normalized feature value by not AKI patients in the column “not AKI.” We show the subtracted result in column “Delta” from column “AKI” to “not AKI.” PTT, partial thromboplastin time; INR, international normalized ratio; PT, prothrombin time; BUN, blood urea nitrogen; WBC, white blood count; SpO_2_, blood oxygen saturation; SBP, systolic blood pressure; DBP, diastolic blood pressure; RR, respiration rate; MBP, mean arterial blood pressure*.

Patients' comorbidities and medications data were also analyzed by AKI case and not AKI case. From Table 5, we find that with comorbidities such as cirrhosis, coronary artery disease (CAD), or congestive heart failure, the AKI percentage is more than 50%, while it is near to 50% with liver disease and diabetes. These comorbidities may affect patients to develop AKI.

**Table 5:** Analysis of the comorbidities data.

**Comorbidity**	**AKI**	**Not AKI**	**Ratio: AKI/(AKI+not AKI) (%)**	**Ratio: not AKI/(AKI+not AKI) (%)**
Cirrhosis	70	49	58.82	41.18
CAD	1,325	931	58.73	41.27
CHF	602	567	51.5	48.5
Liver disease	20	21	48.78	51.22
Diabetes	24	28	46.15	53.85
MI	457	653	41.17	58.83
Hypertension	51	83	38.06	61.94
Jaundice	24	42	36.36	63.64
PV	15	30	33.33	66.67
UTI	87	183	32.22	67.78
Sleep apnea	1	7	12.5	87.5

*The value in column “AKI” is the total number of the patients who developed AKI with the specified comorbidity, while the “not AKI” column shows the total number of patients without the AKI case. CAD, coronary artery disease; CHF, congestive heart failure; MI, myocardial infarction; PV, peripheral vascular; UTI, urinary tract infection*.

For medication, more than 50% patients who had the medications such as drugs used in diabetes or diuretics developed AKI (shown in Table 6), which may show the probable positive association between AKI and these features. For medications such as lipid-lowering medication, antithrombotic agents, and agents acting on the renin–angiotensin system, <50% of the patients developed AKI, which may mean that these medications can prevent AKI onset to some extent.

**Table 6:** Analysis of the medications data.

**Medication**	**AKI**	**Not AKI**	**Ratio: AKI/(AKI+not AKI) (%)**	**Ratio: not AKI/(AKI+not AKI) (%)**
A10	3,667	3,064	54.48	45.52
C03	4,249	3,780	52.92	47.08
C02	2,092	2,396	46.61	53.39
M01	1,147	1,342	46.08	53.92
C10	2,994	3,635	45.17	54.83
B01	7,180	10,288	41.10	58.90
C09	1,067	2,038	34.36	65.64

*The value in column “AKI” is the total number of the patients who developed AKI with the specified medication, while the “not AKI” column shows the total number of patients without AKI case. C03, diuretics; M01, nonsteroidal anti-inflammatory drugs; V08, contrast media; C09, agents acting on the renin–angiotensin system; C02, antihypertensives; A10, drugs used in diabetes; B01, antithrombotic agents; C10, lipid-lowering medication*.

#### Impact Weight Analysis

**Figure 9:**
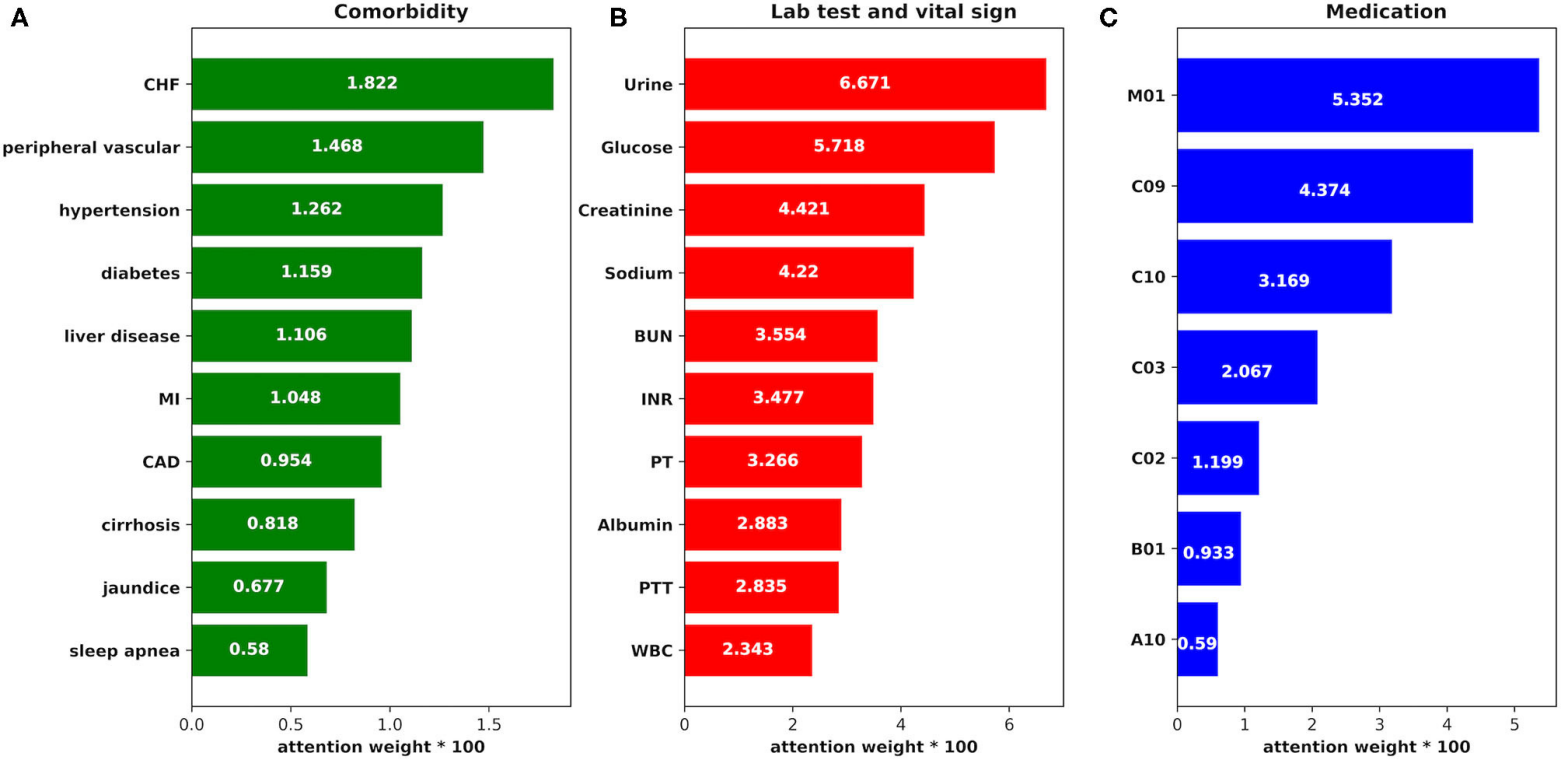
The attention weight of the features. **(A)** The attention weight of each comorbidity to AKI case. CHF, congestive heart failure; MI, myocardial infarction; CAD, coronary artery disease. **(B)** The attention weight of each item in the lab tests and vital signs to AKI case. BUN, blood urea nitrogen; INR, international normalized ratio; PT, prothrombin time; PTT, partial thromboplastin time; WBC, white blood count. **(C)** The attention weight of each medication to AKI case. M01, nonsteroidal anti-inflammatory drugs; C09, agents acting on the renin–angiotensin system; C10, lipid-lowering medication; C03, diuretics; C02, antihypertensives; B01, antithrombotic agents; A10, drugs used in diabetes.

To explore how the features in our model impact the AKI prediction result, we employed attention function as one part of the prediction model. We got the weight parameters produced by attention function which may refer to the impact of the features to AKI result, and the weight data are shown in Figure 9 (we chose the top impact features by weight from largest to smallest in each group: comorbidity, medication, and lab test and vital sign).

From the figure, we find that:

1) The weight of feature urine is the largest, which is consistent with previous research results (40), suggesting that clinicians should pay more attention to the urine volume value of patients during treatment to detect AKI as early as possible.
2) The risk of AKI is significantly correlated with blood glucose value, which is consistent with a study on the influence of perioperative blood glucose level on the prognosis of hospitalization in patients undergoing coronary bypass surgery in Imran (41). Hyperglycemia may induce the accumulation of oxidative products at the mitochondrial level, which may damage the renal endothelial cells (42). Therefore, blood glucose control in severe patients is important in the prevention and treatment of AKI.
3) Creatinine and BUN are traditional indicators of renal function. In the proposed model, the weights of creatinine and BUN are also relatively high, suggesting that these features are meaningful to AKI prediction, which is consistent with the results in previous studies (43).
4) The feature sodium may also become an important parameter for the prediction according to its high weight in the prediction, which is consistent with Lombardi's research results (44). The change in sodium level leads to a change of osmotic pressure and AKI may develop finally.
5) Since non-steroidal anti-inflammatory drugs (NSAIDs) can inhibit prostaglandin synthesis, which may develop renal arteriole contraction, renal blood perfusion reduction, and AKI (45), it gets the highest weight in the “medication” group.
6) Lipid-lowering medication may be an important indicator of AKI prediction according to its weight on the figure. It has been reported that these medications can prevent AKI onset in coronary bypass surgery (46), so AKI risk may be reduced among those ICU patients who have lipid-lowering medications during treatment.
7) In the group “comorbidity,” congestive heart failure, peripheral vascular, hypertension, and diabetes are the top four comorbidities that impact AKI prediction, which may demonstrate that these comorbidities affect renal function.

## Discussion

In our study, an attention-based neural network approach was proposed for the prediction of AKI risk and for the analysis of the impact of medical features. The approach was trained by the MIMIC-III dataset and applied in clinical practice.

Since AKI is one of the high-incidence diseases among hospitalized patients in the ICU, AKI case information is important for clinicians to make preliminary diagnosis and decision. Besides, because an increase in the severity of AKI is related to an increase in mortality (2), AKI stage information may help to better learn about AKI severity and provide intervention that may be prompt and effective, to reduce mortality. Furthermore, since the KDIGO criteria are referred to define the AKI case and stages in our study, there are several time points used for the definition, such as 24, 48 h, and 7 days, and the onset time interval is set up according to these points. All these factors impel us to provide an approach that can predict the information of AKI case, AKI stage, and AKI onset time interval.

In the AKI prediction approach, we firstly developed a temporal convolutional network for future value prediction of the temporal features by their historical records, such as laboratory test results and vital signs, during the ICU stay; then we employed the attention-based neural network model which combines the predicted future data together with the patients' other selected features data, such as demographics, medications, and comorbidities, as the input sequence, to predict AKI case, AKI stage, and AKI onset time interval. Compared with the traditional machine learning algorithms, the LSTM model, and the recent AKI prediction models such as MN+HieLSTM (14), our approach improved the performance of AKI case prediction on MIMIC-III dataset by around 5% AUC.

We also had a comparison between the two methods—attention-based neural network with predicted future value and that without predicted future value—in the three experimental cases of our study. The results reflected that the model with predicted future value improved the prediction performance by around 4% AUC. The possible explanation is that the temporal features play important roles in the prediction and the predicted future value of each temporal feature can better represent the trend of the patient's AKI risk than its historical value.

For the AKI stage prediction in three experimental cases, the AUC value increased with the AKI stage, which means that the higher the AKI stage, the better the model performance. This result is likely to be related to AKI stage definition by the KDIGO criteria (details in section AKI Definition): the stage depends on the value of serum creatinine and that of urine volume mainly. The higher the serum creatinine value, the higher the AKI stage, and there may be positive associations between serum creatinine and other features, such as the laboratory test results and vital signs, which make the features of the high AKI stage easier to be recognized by the proposed model.

From the results of AKI onset time interval prediction, we found that the model performance in the first two intervals was better than in the other intervals, and this may indicate that with our model, the patients' selected features data can only affect the prediction result within the next 48 h, and the influence may consistently decline over time. To make a more accurate onset interval prediction, we set the length of each onset interval to 12 h and focus on the first 24 h of the prediction interval in experimental case 1. The performance in the first onset interval was better than in the second one (68.9% AUC compared with 67.5% AUC), but they were both lower than the performance in the first onset interval of case 2 and case 3 (around 73% AUC), which may show that prediction performance decreases as the length of onset interval decreases.

To validate the performance of our approach, we applied it in a real clinical AKI prediction. During the model implementation in clinical practice, the prediction performance decreased by a varying degree. The decline may due to a discrepancy of the medical feature values made by different physicians, such as the diagnosis of comorbidities; besides, the values produced by different medical equipment may also be different.

From the analysis result of the association between medical features and AKI case, we found that features, such as lactate, glucose, creatinine, blood urea nitrogen, prothrombin time, and partial thromboplastin time, may have positive associations with the AKI case, while another group, such as platelet, hemoglobin, hematocrit, urine, and INR, may potentially be inversely associated with the AKI case.

Furthermore, with the help of the attention function in the prediction model, we identified the impact weights of the selected medical features in our study. The features were classified into three groups: “medication,” “comorbidity,” and “lab test and vital sign.” From the attention weights in Figure 9, we found that features in the group “lab test and vital sign” played more important roles in AKI prediction than features in the other two groups, especially the features urine, glucose, creatinine, sodium, and blood urea nitrogen. Moreover, the influence of features in the group “medication” to AKI cannot be ignored according to their high attention weights. As the top three impact features in the group “medication,” non-steroidal anti-inflammatory drugs, agents acting on the renin–angiotensin system, and lipid-lowering medication show their importance to the AKI results.

There are two main limitations in our study:

1) The data we used for the model training were the EHR records during the first 24 (48) h of the new admitted ICU patients, which may lose some significant information that can help improve the performance of AKI prediction, such as past medical history, past laboratory test results, and past medications of the patients before their ICU admission.
2) We did not consider the intervention data as the input of the model during the observation interval, and this could potentially bias the prediction results since the interventions taken for patients during their ICU stays may affect the clinical outcomes.

In conclusion, we proposed an end-to-end AKI prediction approach in our study. The model takes the patients' EHR data during their ICU admission as the input and the AKI prediction result as the output, which provides a convenient and efficient means for the clinicians to evaluate the comprehensive AKI information, including the AKI risk, the possible severity, and onset time interval, for the new admitted ICU patients by their clinical data and take the appropriate interventions before the onset of AKI. Besides, we further explore the association between the features and the AKI onset and the impact of each feature to the AKI result, which may help clinicians to better observe the features that have a great influence on AKI risk, and take targeted treatments to keep values of the features in normal ranges.

AKI is a dangerous and complicated disease with a potentially life-threatening condition. To avoid physical injury caused by AKI, in the future, we may improve our approach from the following aspects:

1) More training data for the prediction model: patients' medical imaging data, such as the color ultrasound of the kidney, will be included in the training dataset, to get more medical information.
2) AKI prediction model for a specific disease group: since the roles of medical features may vary in different disease groups, and a general AKI prediction model may not fit well for all the groups, we may develop a new AKI prediction model which is based on the proposed approach and is integrated with the knowledge ontology of the targeted disease group.
3) Develop efficient methods to identify the abnormal renal decline as early as possible: to detect the AKI risk of patients before they are admitted to the ICU, we will consider more medical data from the patients, including their outpatient data, inpatient data, and physical examination data, to track the temporal values of the features which have a great influence on AKI risk and explore patterns predictive of renal decline that may make detection of early AKI risk feasible.

## Data Availability Statement

Publicly available datasets were analyzed in this study. This data can be found at: https://physionet.org/content/mimiciii/1.4/.

## Author Contributions

ZC, MC, and XL designed the studies, built and performed the neural network, and wrote the manuscript. XS and XG contributed to the result analysis and data interpretation. QL, YZ, LW, YH, YL, JX, and YF contributed to the EHR data preparation and the medical conception description. All authors contributed to manuscript revision and approved the submitted version.

## Conflict of Interest

ZC was employed by company Fujian Province Lianpu Network Technology Co., Ltd. The remaining authors declare that the research was conducted in the absence of any commercial or financial relationships that could be construed as a potential conflict of interest.

## References

[1] Mercado MG Smith DK Guard EL. Acute kidney injury: diagnosis and management. Am Fam Phys. (2019) 100:687–94. 31790176

[2] Epidemiology of acute kidney injury in critically ill patients: the multinational AKI-EPI study. Intens. Care Med. (2015). 41:1411–23. 10.1007/s00134-015-3934-7 26162677

[3] Zeng X Mcmahon GM Brunelli SM Bates DW Waikar SS. Incidence, outcomes, and comparisons across definitions of AKI in hospitalized individuals. Clin J Am Soc Nephrol. (2014). 9:12–20. 10.2215/CJN.02730313 24178971 PMC3878695

[4] Kate RJ Perez RM Mazumdar D Pasupathy KS Nilakantan V. Prediction and detection models for acute kidney injury in hospitalized older adults. BMC Med Inform Decis Mak. (2016). 16:1–11. 10.1186/s12911-016-0277-4 27025458 PMC4812614

[5] Rough K Dai AM Zhang K Xue Y Vardoulakis LM Cui C. Predicting inpatient medication orders from electronic health record data. Clin Pharmacol Ther. (2020). 108:145–54. 10.1002/cpt.1826 32141068 PMC7325318

[6] Yang Y Xie P Gao X Cheng C Li C Zhang H Xing E. Predicting discharge medications at admission time based on deep learning. (2017) *arXiv:1-12*.

[7] Miotto R Li L Kidd BA Dudley JT. Deep patient: an unsupervised representation to predict the future of patients from the electronic health records. Sci Rep. (2016). 6:1–10. 10.1038/srep26094 27185194 PMC4869115

[8] Darabi S Kachuee M Fazeli S Sarrafzadeh M. TAPER: Time-aware patient EHR representation. IEEE J Biomed Heal Informatics. (2020). 24:3268–75. 10.1109/JBHI.2020.2984931 32287023

[9] Choi E Schuetz A Stewart WF Sun J. Using recurrent neural network models for early detection of heart failure onset. J Am Med Informatics Assoc. (2017). 24:361–70. 10.1093/jamia/ocw112 27521897 PMC5391725

[10] Nguyen P Tran T Wickramasinghe N Venkatesh S. Deepr: a convolutional net for medical records. IEEE J Biomed Heal Informatics. (2017). 21:22–30. 10.1109/JBHI.2016.2633963 27913366

[11] Li Y Yao L Mao C Srivastava A Jiang X Luo Y. Early prediction of acute kidney injury in critical care setting using clinical notes. In: Proc - 2018 IEEE Int Conf Bioinforma Biomed BIBM. Chicago, IL (2019). p. 683–6. 10.1109/BIBM.2018.8621574 PMC7768909 33376624

[12] Mihalcea R Liu H Lieberman H. NLP (Natural Language Processing) for NLP (Natural Language Programming). In: Computational Linguistics and Intelligent Text Processing, 7th International Conference, CICLing 2006. Mexico City (2006). 10.1007/11671299_34

[13] Tomašev N Glorot X Rae JW Zielinski M Askham H Saraiva A. A clinically applicable approach to continuous prediction of future acute kidney injury. Nature. (2019). 572:116–9. 10.1038/s41586-019-1390-1 31367026 PMC6722431

[14] Xu Z Chou J Zhang XS Luo Y Isakova T Adekkanattu P. Identifying sub-phenotypes of acute kidney injury using structured and unstructured electronic health record data with memory networks. J Biomed Inform. (2020). 102:103361. 10.1016/j.jbi.2019.103361 31911172

[15] Koyner JL Carey KA Edelson DP Churpek MM. The development of a machine learning inpatient acute kidney injury prediction model. Crit Care Med. (2018). 46:1070–7. 10.1097/CCM.0000000000003123 29596073

[16] Bellomo R Ronco C Kellum JA Mehta RL Palevsky P. Acute renal failure - definition, outcome measures, animal models, fluid therapy and information technology needs: the Second International Consensus Conference of the Acute Dialysis Quality Initiative (ADQI) Group. Crit Care. (2004). 8:R204. 10.1186/cc2872 15312219 PMC522841

[17] Akcan-Arikan A Zappitelli M Loftis LL Washburn KK Jefferson LS Goldstein SL. Modified RIFLE criteria in critically ill children with acute kidney injury. Kidney Int. (2007). 71:1028–35. 10.1038/sj.ki.5002231 17396113

[18] Pickering JW Endre ZH. GFR shot by RIFLE: errors in staging acute kidney injury. Lancet. (2009). 373:1318–9. 10.1016/S0140-6736(09)60751-0 19376434

[19] Kellum JA Lameire N Aspelin P Barsoum RS Burdmann EA Goldstein SL. Kidney disease:. KDIGO clinical practice guideline for acute kidney injury. Kidney Int Suppl. (2012) 2:1–138. 10.1159/000339789

[20] Levey A Coresh J Greene T Inker L Hendriksen S Kusek J. Using standardized serum creatinine values in the modification of diet in renal disease study equation for estimating glomerular filtration rate. Ann Intern Med. (2006). 145:247–54. 10.7326/0003-4819-145-4-200608150-00004 16908915

[21] Johnson AEW Pollard TJ Shen L Lehman LH Feng M Ghassemi M. MIMIC-III, a freely accessible critical care database. Sci Data. (2016). 3:160035. 10.1038/sdata.2016.35 27219127 PMC4878278

[22] Chen L Lu J Zhang N Huang T Cai Y-D. A hybrid method for prediction and repositioning of drug Anatomical Therapeutic Chemical classes. Mol Biosyst. (2014). 10:868–77. 10.1039/c3mb70490d 24492783

[23] Guo JJ Diehl MC Felkey BG Gibson JT Barker KN. Comparison and analysis of the national drug code systems among drug information databases. Drug Inf Assoc. (1998). 32:769–75. 10.1177/009286159803200317

[24] Kirsch I Deacon BJ Huedo-Medina TB Scoboria A Moore TJ Johnson BT. Initial severity and antidepressant benefits: a meta-analysis of data submitted to the food and drug administration. PLoS Med. (2008). 5:e45. 10.1371/journal.pmed.0050045 18303940 PMC2253608

[25] He Y Zhao J. Temporal convolutional networks for anomaly detection in time series. J Phys Conf Ser. (2019). 1213:042050. 10.1088/1742-6596/1213/4/042050

[26] Bai S Kolter JZ Koltun V. An empirical evaluation of generic convolutional and recurrent networks for sequence modeling. (2018). arXiv:1803.01271.

[27] Kok C Jahmunah V Oh SL Zhou X Gururajan R Tao X. Automated prediction of sepsis using temporal convolutional network. Comput Biol Med. (2020). 2020:103957. 10.1016/j.compbiomed.2020.103957 32938540

[28] Razavian N Sontag D. Temporal Convolutional Neural Networks for Diagnosis from Lab Tests. (2015). Available online at: http://arxiv.org/abs/1511.07938

[29] Vaswani A Shazeer N Parmar N Uszkoreit J Jones L Gomez AN. Attention is all you need. Adv Neural Inf Process Syst. (2017). 2017:5999–6009.

[30] Hochreiter S Schmidhuber J. Long short-term memory. Neural Comput. (1997). 9:1735–80. 10.1162/neco.1997.9.8.1735 9377276

[31] Ba J Frey B. Adaptive dropout for training deep neural networks. In: Advances in Neural Information Processing Systems. (2013). Available online at: https://proceedings.neurips.cc/paper/2013/file/7b5b23f4aadf9513306bcd59afb6e4c9-Paper.pdf

[32] Li Y Yuan Y. Convergence analysis of two-layer neural networks with ReLU Activation. In: Advances in Neural Information Processing Systems. Princeton, NJ (2017). p. 597–607. Available online at: https://proceedings.neurips.cc/paper/2017/file/a96b65a721e561e1e3de768ac819ffbb-Paper.pdf

[33] Alizadeh M Fernández-Marqués J Lane ND Gal Y. A empirical study of binary neural networks' optimisation. In: International Conference on Learning Representations. Oxford (2018). Available online at: https://openreview.net/forum?id=rJfUCoR5KX

[34] Hubáček O Šourek G Železný F. Learning to predict soccer results from relational data with gradient boosted trees. Mach Learn. (2019). 108:29–47. 10.1007/s10994-018-5704-6

[35] DeMaris A. A tutorial in logistic regression. J Marriage Fam. (1995). 57:956–68. 10.2307/353415

[36] Pal M. Random forest classifier for remote sensing classification. Int J Remote Sens. (2005). 26:217–22. 10.1080/01431160412331269698

[37] Lipton ZC Kale DC Elkan C Wetzel R. Learning to diagnose with LSTM recurrent neural networks. In: 4th International Conference on Learn Represent ICLR 2016 - Conf Track Proceedings. Pittsburgh, PA (2016). p. 1–18.

[38] Abadi M Barham P Chen J Chen Z Davis A Dean J. TensorFlow: a system for large-scale machine learning. In: 12th {USENIX} Symposium on Operating Systems Design and Implementation ({OSDI} 16). Savannah, GA: {USENIX} Association (2016). p. 265–83. Available online at: https://www.usenix.org/conference/osdi16/technical-sessions/presentation/abadi

[39] Buitinck L Louppe G Blondel M Pedregosa F Mueller A Grisel O. {API} design for machine learning software: experiences from the SCIKIT-learn project. CoRR. (2013). abs/1309.0.

[40] Kellum JA Sileanu FE Murugan R Lucko N Shaw AD Clermont G. Classifying AKI by urine output versus serum creatinine level. J Am Soc Nephrol. (2015). 26:2231–38. 10.1681/ASN.2014070724 25568178 PMC4552117

[41] Imran SA Ransom TPP Buth KJ Clayton D Al-Shehri B Ur E. Impact of admission serum glucose level on in-hospital outcomes following coronary artery bypass grafting surgery. Can J Cardiol. (2010). 26:151–4. 10.1016/S0828-282X(10)70357-3 20352135 PMC2851469

[42] Siegelaar SE Kulik W Lenthe H Van Mukherjee R Hoekstra JBL Devries JH. A randomized clinical trial comparing the effect of basal insulin and inhaled mealtime insulin on glucose variability and oxidative stress. Diabetes Obes Metab. (2010). 11:709–14. 10.1111/j.1463-1326.2009.01037.x 19320663

[43] Rodríguez E Soler MJ Rap O Barrios C Orfila MA Pascual J. Risk factors for acute kidney injury in severe rhabdomyolysis. PLoS ONE. (2013). 8:e82992. 10.1371/journal.pone.0082992 24367578 PMC3867454

[44] Lombardi G Ferraro PM Naticchia A Gambaro G. Serum sodium variability and acute kidney injury: a retrospective observational cohort study on a hospitalized population. Intern Emerg Med. (2020) 16:617–24. 10.1007/s11739-020-02462-5 32776204 PMC8049924

[45] Vogt L Laverman GD Navis G. Time for a comeback of NSAIDs in proteinuric chronic kidney disease? Neth J Med. (2010). 68:400–7. 21209465

[46] Lewicki M Ng I Schneider A. HMG CoA reductase inhibitors (statins) for preventing acute kidney injury after surgical procedures requiring cardiac bypass: a systematic review and meta-analysis. Nephrology. (2014). 19:30. 10.1002/14651858.CD010480 PMC10788137 25758322

