# Supplementary material for: Analysis of the Impact of Medical Features and Risk Prediction of Acute Kidney Injury for Critical Patients Using Temporal Electronic Health Record Data With Attention-Based Neural Network
Source: Front Med (Lausanne). 2021 Jun 4;8:658665. doi: 10.3389/fmed.2021.658665 (PMC8212017; doi:10.3389/fmed.2021.658665)
Supplement: Supplementary file 1 [file Table_1.DOCX]

Supplementary Material

# Supplementary Figures and Tables

## Supplementary Tables

**Supplementary Table 1.** AKI stage prediction: the performance of the methods applied in experimental case 3.

| **Experimental case** | **Methods** | **Specificity** | **Sensitivity** | **AUC** |
| --- | --- | --- | --- | --- |
| **Stage 1** | **AM with PFV** | **0.5416 ± 0.0119** | **0.7139 ± 0.0107** | **0.6742 ± 0.0097** |
| **Stage 1** | AM without PFV | 0.5142 ± 0.0132 | 0.7002 ± 0.0149 | 0.6501 ± 0.0079 |
| **Stage 1** | LSTM | 0.4918 ± 0.0201 | 0.6712 ± 0.0121 | 0.6331 ± 0.0130 |
| **Stage 1** | GBTs | 0.4613 ± 0.0193 | 0.6789 ± 0.0180 | 0.6138 ± 0.0045 |
| **Stage 1** | RF | 0.4508 ± 0.0150 | 0.6598 ± 0.0178 | 0.5949 ± 0.0107 |
| **Stage 1** | LR | 0.4223 ± 0.0128 | 0.6341 ± 0.0190 | 0.5791 ± 0.0108 |
| **Stage 2** | **AM with PFV** | **0.5837 ± 0.0143** | **0.8083 ± 0.0128** | **0.7655 ± 0.0130** |
| **Stage 2** | AM without PFV | 0.5604 ± 0.0152 | 0.7791 ± 0.0134 | 0.7498 ± 0.0122 |
| **Stage 2** | LSTM | 0.5370 ± 0.0124 | 0.7478 ± 0.0131 | 0.7201 ± 0.0063 |
| **Stage 2** | GBTs | 0.5009± 0.0098 | 0.7061 ± 0.0147 | 0.6889 ± 0.0164 |
| **Stage 2** | RF | 0.4801 ± 0.0134 | 0.6780 ± 0.0126 | 0.6505 ± 0.0056 |
| **Stage 2** | LR | 0.4530 ± 0.0101 | 0.6544 ± 0.0166 | 0.6234 ± 0.0149 |
| **Stage 3** | **AM with PFV** | **0.7302 ± 0.0149** | **0.7751 ± 0.0139** | **0.8279 ± 0.0081** |
| **Stage 3** | AM without PFV | 0.7128 ± 0.0209 | 0.7502± 0.0125 | 0.8008 ± 0.0019 |
| **Stage 3** | LSTM | 0.6889 ± 0.0133 | 0.7291 ± 0.0128 | 0.7745 ± 0.0150 |
| **Stage 3** | GBTs | 0.6501 ± 0.0188 | 0.6971 ± 0.0164 | 0.7001 ± 0.0113 |
| **Stage 3** | RF | 0.6101 ± 0.0099 | 0.6700 ± 0.0101 | 0.6578 ± 0.0119 |
| **Stage 3** | LR | 0.5987 ± 0.0139 | 0.6491 ± 0.0201 | 0.6399 ± 0.0113 |

AM: Attention Model, PFV: Predicted Future Values by TCN.

**Supplementary Table 2.** AKI onset time interval prediction: the performance of the methods applied in experimental case 3.

| **Experimental case** | **Methods** | **Specificity** | **Sensitivity** | **AUC** |
| --- | --- | --- | --- | --- |
| **0-24h** | **AM with PFV** | **0.5865 ± 0.0116** | **0.7291 ± 0.0091** | **0.7518 ± 0.0131** |
| **0-24h** | AM without PFV | 0.5687 ± 0.0091 | 0.7062 ± 0.0144 | 0.7301 ± 0.0139 |
| **0-24h** | LSTM | 0.5407 ± 0.0092 | 0.6608 ± 0.0109 | 0.7041 ± 0.0140 |
| **0-24h** | GBTs | 0.5139 ± 0.0201 | 0.6499 ± 0.0034 | 0.6631 ± 0.0181 |
| **0-24h** | RF | 0.4998 ± 0.0105 | 0.6333 ± 0.0088 | 0.6582 ± 0.0130 |
| **0-24h** | LR | 0.4829 ± 0.0107 | 0.6202 ± 0.0068 | 0.6477 ± 0.0091 |
| **24-48h** | **AM with PFV** | **0.5448 ± 0.0128** | **0.6890 ± 0.0085** | **0.7091 ± 0.0106** |
| **24-48h** | AM without PFV | 0.5354 ± 0.0046 | 0.6712 ± 0.0021 | 0.6859 ± 0.0101 |
| **24-48h** | LSTM | 0.5104 ± 0.0108 | 0.6523 ± 0.0155 | 0.6698 ± 0.0084 |
| **24-48h** | GBTs | 0.4858± 0.0102 | 0.6303 ± 0.0109 | 0.6438 ± 0.0058 |
| **24-48h** | RF | 0.4696 ± 0.0117 | 0.6131 ± 0.0173 | 0.6230 ± 0.0011 |
| **24-48h** | LR | 0.4533 ± 0.0071 | 0.6005 ± 0.0142 | 0.6099 ± 0.0096 |
| **48-72h** | **AM with PFV** | **0.4028 ± 0.0079** | **0.6190 ± 0.0167** | **0.6371 ± 0.0159** |
| **48-72h** | AM without PFV | 0.3998 ± 0.0107 | 0.6107± 0.0133 | 0.6299 ± 0.0019 |
| **48-72h** | LSTM | 0.3807 ± 0.0078 | 0.6001 ± 0.0029 | 0.6039 ± 0.0105 |
| **48-72h** | GBTs | 0.3551 ± 0.0181 | 0.5839 ± 0.0102 | 0.5801 ± 0.0088 |
| **48-72h** | RF | 0.3371 ± 0.0112 | 0.5680 ± 0.0011 | 0.5679 ± 0.0071 |
| **48-72h** | LR | 0.3263 ± 0.0049 | 0.5550 ± 0.0107 | 0.5595 ± 0.0090 |

AM: Attention Model, PFV: Predicted Future Values by TCN.

(For the performance of AKI onset time interval prediction in the intervals: 72-96h, 96-120h were around 50% AUC, the comparison of the methods was applied in the intervals: 0-24h, 24-48h, 48-72h).

**Supplementary Table 3.** The performance of the future value prediction by TCN model.

| Feature name | RMSE (24h) | RMSE (24h)/mean (%) | RMSE (48h) | RMSE (48h)/mean (%) |
| --- | --- | --- | --- | --- |
| Lactate | 0.1984 | 3.203% | 0.1705 | 3.014% |
| Creatinine | 0.0252 | 2.101% | 0.0229 | 2.018% |
| BUN | 0.6488 | 2.403% | 0.6024 | 2.132% |
| PT | 0.5121 | 3.201% | 0.5009 | 3.019% |
| PTT | 1.2787 | 3.119% | 1.2375 | 3.008% |
| Glucose | 4.4870 | 3.205% | 4.3571 | 3.105% |
| Anion gap | 0.5181 | 3.701% | 0.5281 | 3.791% |
| Potassium | 0.0796 | 1.878% | 0.0782 | 1.861% |
| Bilirubin | 0.0625 | 1.963% | 0.0617 | 1.935% |
| WBC | 0.3495 | 2.879% | 0.3501 | 2.884% |
| Chloride | 3.0460 | 2.901% | 3.0291 | 2.885% |
| Sodium | 3.2305 | 2.341% | 3.2218 | 2.335% |
| Bicarbonate | 0.6176 | 2.617% | 0.6189 | 2.623% |
| Albumin | 0.0619 | 2.031% | 0.0621 | 2.038% |
| Platelet | 7.3934 | 3.504% | 7.3011 | 3.460% |
| Hemoglobin | 0.4404 | 4.101% | 0.4295 | 4.010% |
| Hematocrit | 0.9242 | 2.937% | 0.9229 | 2.923% |
| INR | 0.0489 | 3.101% | 0.0478 | 3.021% |
| SpO2 | 1.8773 | 1.935% | 1.8578 | 1.925% |
| HeartRate | 3.0139 | 3.312% | 2.9801 | 3.255% |
| SBP | 4.1311 | 3.501% | 4.143 | 3.515% |
| MBP | 2.2520 | 2.891% | 2.1902 | 2.802% |
| DBP | 2.0070 | 3.3451% | 2.0970 | 3.489% |
| RR | 0.5501 | 2.918% | 0.5431 | 2.887% |

The value in column ‘RMSE (24h)’ represents the Root Mean Square Error value of the prediction, which takes the first 24h in ICU as the observation interval, for each feature; The value in column ‘RMSE (24h)/mean’ is the result of the value in column ‘RMSE (24h)’ divided by the mean of each feature.

The value in column ‘RMSE (48h)’ represents the Root Mean Square Error value of the prediction, which takes the first 48h in ICU as the observation interval, for each feature; The value in column ‘RMSE (48h)/mean’ is the result of the value in column ‘RMSE (48h)’ divided by the mean of each feature value.

PTT: Partial Thromboplastin Time, INR: International Normalized Ratio, PT: Prothrombin Time, BUN: Blood Urea Nitrogen, WBC: White Blood Count, SpO2: blood oxygen saturation, SBP: Systolic Blood Pressure, DBP: Diastolic Blood Pressure, RR: Respiration Rate, MBP: Mean arterial Blood Pressure.
